# Genetic Predisposition to Myelodysplastic Syndromes: A Challenge for Adult Hematologists

**DOI:** 10.3390/ijms22052525

**Published:** 2021-03-03

**Authors:** Elena Crisà, Paola Boggione, Maura Nicolosi, Abdurraouf Mokhtar Mahmoud, Wael Al Essa, Bassel Awikeh, Anna Aspesi, Annalisa Andorno, Renzo Boldorini, Irma Dianzani, Gianluca Gaidano, Andrea Patriarca

**Affiliations:** 1 Division of Hematology, Department of Translational Medicine, University of Eastern Piedmont and Azienda Ospedaliero-Universitaria Maggiore della Carità, 28100 Novara, Italy; 20032528@studenti.uniupo.it (P.B.); maura.nicolosi@med.uniupo.it (M.N.); abdurraouf.mahmoud@uniupo.it (A.M.M.); wael.alessa@uniupo.it (W.A.E.); bassel.awikeh@uniupo.it (B.A.); andrea.patriarca@med.uniupo.it (A.P.); 2 Laboratory of Genetic Pathology, Division of Pathology, Department of Health Sciences, University of Eastern Piedmont and Azienda Ospedaliero-Universitaria Maggiore della Carità, 28100 Novara, Italy; anna.aspesi@med.uniupo.it (A.A.); irma.dianzani@med.uniupo.it (I.D.); 3 Division of Pathology, Department of Health Sciences, University of Eastern Piedmont and Azienda Ospedaliero-Universitaria Maggiore della Carità, 28100 Novara, Italy; annalisa.andorno@maggioreosp.novara.it (A.A.); renzo.boldorini@med.uniupo.it (R.B.)

**Keywords:** genetic predisposition, myelodysplastic syndromes, inherited bone marrow failure

## Abstract

Myelodysplastic syndromes (MDS) arising in the context of inherited bone marrow failure syndromes (IBMFS) differ in terms of prognosis and treatment strategy compared to MDS occurring in the adult population without an inherited genetic predisposition. The main molecular pathways affected in IBMFS involve telomere maintenance, DNA repair, biogenesis of ribosomes, control of proliferation and others. The increased knowledge on the genes involved in MDS pathogenesis and the wider availability of molecular diagnostic assessment have led to an improvement in the detection of IBMFS genetic predisposition in MDS patients. A punctual recognition of these disorders implies a strict surveillance of the patient in order to detect early signs of progression and promptly offer allogeneic hematopoietic stem cell transplantation, which is the only curative treatment. Moreover, identifying an inherited mutation allows the screening and counseling of family members and directs the choice of donors in case of need for transplantation. Here we provide an overview of the most recent data on MDS with genetic predisposition highlighting the main steps of the diagnostic and therapeutic management. In order to highlight the pitfalls of detecting IBMFS in adults, we report the case of a 27-year-old man affected by MDS with an underlying telomeropathy.

## 1. Introduction

Myelodysplastic syndromes (MDS) comprise a group of clonal hematopoietic stem cell disorders characterized by ineffective hematopoiesis, one or more peripheral cytopenias, single or multilineage dysplasia in the bone marrow (BM), and an increased risk of progressing to acute myeloid leukemia (AML), occurring eventually in approximately 30% of the cases, more rapidly in higher risk patients [1,2]. MDS usually arise in the elderly, with a median age at diagnosis of 72–75 years, in a context of somatic mutations acquired during aging [3,4,5]. These mutations target a limited number of cellular processes, including RNA splicing, epigenetic and transcriptional regulation, as well as signal transduction pathways involved in cell growth and differentiation. The sequential accumulation of oncogenic hits drives disease evolution from asymptomatic clonal hematopoiesis to frank MDS, and, ultimately, to secondary AML [6].

The annual incidence of MDS in the adult population is about 4–5 per 100,000, increasing to 50 per 100,000 in individuals over 70 years [7,8,9]. By contrast, MDS presenting in children or younger adults are rare, with an annual incidence of 1–4 cases per million, and many occur in the context of genetic predisposition or inherited BM failure syndromes (IBMFS) [10]. Currently, the increased knowledge on the genes involved in MDS pathogenesis, and the wider availability of molecular diagnostic tests based on next generation sequencing analysis (NGS), is leading to an increase in the detection of genetic predisposition in MDS patients. The initial recognition of IBMFS is dependent upon the patient’s clinical and physical features together with the hematologic findings, and it is then confirmed by genetic testing [11]. However, occasionally IBMFS patients have no physical alterations and the only clue for an inherited gene alteration predisposing to MDS is the young age or the family history. Recognizing the genetic predisposition to MDS may change not only the prognostication of the patient (e.g., higher risk of evolution to AML), but also the treatment strategy, as exemplified by the contraindication to alkylating drugs in transplant conditioning regimen [12].

**Figure 1:**
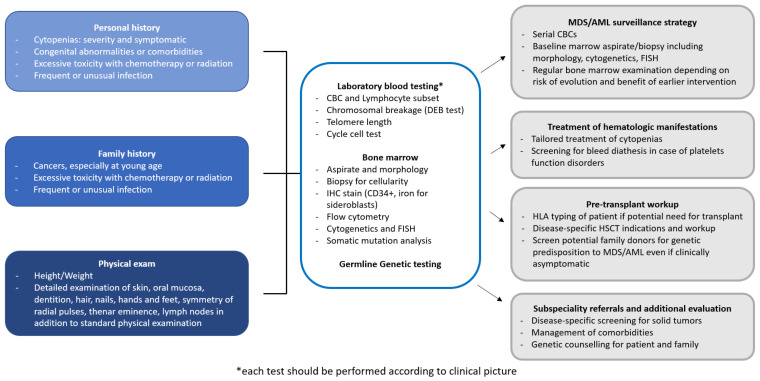
Diagnostic workup of a patient with suspected MDS predisposition syndrome. The diagnostic workup of a patient with MDS predisposition syndrome includes personal and family history, physical examination, and laboratory testing. Identification of a MDS predisposition syndrome has several implications for clinical management, concerning surveillance, treatment choice and pre-transplant workup in case of progression requiring therapy, and subspecialty referrals. AML, Acute myeloid leukemia; CBC, Complete blood count; FISH, Fluorescent in situ hybridization; ICH, Immunohistochemistry; HLA, Human leukocyte antigen; HSCT, Hematopoietic stem cell transplantation; MDS, Myelodysplastic syndromes.

MDS predisposition syndromes may be associated with pancytopenia (telomeropathies, *GATA2* related disorders, Shwachman-Diamond syndrome and *SAMD9/SAMD9L* related disorders), red cell aplasia (Diamond-Blackfan anemia), thrombocytopenia (*RUNX1*, *ANKRD26* and *ETV6* related disorders) and neutropenia (severe congenital neutropenia), which may present not only in children, but also in adults. Each pathologic entity is quite rare, but altogether they account for 4 to 15% of all MDS cases [11,13,14,15,16]. Table 1 summarizes the most common germline disorders associated with MDS, however, there are further rare syndromes not described in this review and the landscape of genetic alterations leading to MDS is increasing day by day [17]. Figure 1 shows a possible workup of a patient with suspected MDS predisposition syndrome and the management implications.

Here we review the most recent data on diagnosis and therapy of MDS with genetic predisposition, and present a case report of a young adult affected by MDS with an underlying telomeropathy that highlights the pitfalls of detecting IBMFS in adult patients, in particular when clear syndromic features are missing or genetic tests taken singularly are not conclusive.

## 2. Telomeropathies

In each cell division, a physiological loss of a certain number of telomeric sequences is unavoidable; however, two biological systems reduce the DNA loss at the chromosome ends: (i) alternative lengthening of telomeres, by which DNA sequences are copied from one telomere to another [55,56]; (ii) telomerase complex, that consists of ribonucleoproteins composed of a main catalytic subunit (hTERT) and an RNA (hTR) that acts as a primer for the addition of telomeric sequences at the DNA 3′ end. Different accessory proteins are needed for the normal function of telomerases, namely dyskeratin (DKC1), NHP2, NOP10, Pontin/Reptin, TCAB1 and GAR1 [57,58,59].

Telomeropathies comprise a wide variety of infrequent diseases caused by genetic defects in telomere maintenance mechanisms or DNA damage response system [60]. Telomeric DNA strictly requires the function of telomerase to maintain the integrity of its sequence. If such protection fails, chromosome end-to-end fusions might be generated, triggering unbalanced chromosome rearrangements [61]. In addition to telomerases, the integrity of telomeres requires also other molecules that are encoded by genes under the control of the DNA damage response. DNA damage triggers the DNA repair machinery or apoptosis, depending on the extent of damage and physiological context. If the DNA damage response system fails to repair the DNA damage, ATM (Ataxia Telangiectasia mutated) and ATR (Ataxia Telangiectasia and Rad3 related) kinase signaling leads to cell cycle arrest and apoptosis through activation of the p53/p21 [62].

Based on the molecular biology of the disease, telomeropathies are classified in: (i) primary telomeropathies, that are the direct expression of telomerase dysfunction; and (ii) secondary telomeropathies, related to the misfunction of the DNA damage response proteins [19,63,64,65,66,67,68].

### 2.1. Primary Telomeropathies

Dyskeratosis congenita (DC) is a complex disease due to mutations in 11 different genes. Three inheritance patterns have been recognized: (i) an X-linked recessive variant targeting the DKC1 gene [18]; (ii) an autosomal dominant presentation linked to mutations in either the *hTR, TERT, TINF2, DKC1* or *ACD* genes; (iii) and, finally, an autosomal recessive DC type, in which mutations can be found in the *TERT, NHP2, NOP10, WRAP53, NOLA3, TCB1, RTEL1, CTC1* and *PARN* genes [23].

Patients with DC are asymptomatic at birth and develop the characteristic triad of mucocutaneous features in the first decade of life. Thereafter, BM failure (BMF) and complications in other organs, such as lung and liver fibrosis, secondary cancer and hematologic malignancies predominate, being the main cause of death in DC [19,20,21,22]. In particular, the risk of developing MDS or AML is markedly greater than in the general population, with a prevalence of 13% in non-transplant patients [22]. Similarly, the frequency of clonal hematopoiesis is significantly higher in individuals with DC as well as in all the other telomere biology disorders (TBD). However, the frequency and the type of genetic alterations in this context is completely different from that reported in sporadic clonal hematopoiesis. In particular, the most frequently mutated genes in sporadic cases, such as *DNMT3A, TET2, ASXL1*, are rarely affected in DC [69,70]. Instead, clonal hematopoiesis in DC occurs without driver mutations and usually harbors a skewed X chromosome inactivation (in females), somatic copy number alterations, or non-recurrent somatic point mutations of the *ASXL1* and *U2AF1* genes [70]. Remarkably, clonal hematopoiesis in DC compensates (through enhanced expression of the normal allele) or reverts (through the deletion of the mutated allele) the genotype of the congenital disease [69,71,72]. More recently, in a cohort of 199 DC with germline *TERT* mutations, 5% were found to have clonal hematopoiesis marked by somatic mutations in the *TERT* promoter. All mutations were localized on the normal *TERT* allele and resulted in enhanced transcription and expression of the normal gene compared to the pathological allele [72]. In conclusion, clonal hematopoiesis in DC ameliorates the phenotype, at least in the hematopoietic stem cell (HSC), leading to the partial improvement of the hematological features in the older population with DC.

On the contrary, the molecular steps leading to acute leukaemia and MDS in DC are less well understood and may differ from those described in clonal hematopoiesis. Recently, a registry study on the outcome of MDS post-allogenic transplantation has revealed a significant higher frequency of *TP53* and *PPM1D* mutations in genetic carriers of telomeropathies [73]. Both these genes are part of the DNA damage response system, which is hyperactive in DC as a consequence of the increased activity of the senescence pathway [73,74,75,76,77,78].

### 2.2. Secondary Telomeropathies

Fanconi anemia (FA) is primarily an autosomal recessive disease (except FANCB which is X-linked), and results from a mutation in one of the 17 genes of the FANC cluster, whose gene products are directly involved in the DNA damage response [28]. Even though the majority of FA patients display a variety of physical abnormalities, including short stature, café au lait spots and hyper/hypopigmentation, abnormal thumbs, absent radii, microcephaly, micro-ophthalmia, and structural renal anomalies, many cases are not diagnosed until the development of aplastic anemia, MDS or AML [24,25]. The diagnostic test for FA is the detection of increased chromosomal breakages in peripheral blood T lymphocytes cultured in the presence of a clastogenic agent [79]. This assay is very sensitive and specific with limitation only in cases with hematopoietic somatic mosaicism, in whom an HSC may have undergone a genetic correction by several mechanisms, leading to marrow and blood cells that have a selective growth advantage. Testing of skin fibroblasts may be required in such cases in order to reach a correct diagnosis [24]. Moreover, even though FA represents a significant risk factor for cancer development, as expected by the function of the involved genes, the actual risk may vary according to the severity of DNA damage response impairment. A recent meta-analysis from the National Institute of Health identified a *FANCD1/BRCA2* double mutated subset that showed a severe impairment of DNA damage response resulting in a cumulative incidence of cancer of 97% by age 7 years, including AML [25,27]. On the contrary, in all the other genotypes (FANCA through FANCQ), more than half of the subjects are symptomatic for BMF with 20% developing AML, 40% MDS, and 30% solid tumors, by age 40 [25,26,27].

From a therapeutic standpoint, androgens represent the only non-transplant treatment approach. Overall, more than 70% of patients reach a response with hematological improvement [80]. Hematopoietic stem cell transplantation (HSCT) is the treatment of choice in case of symptomatic BMF, AML or MDS [81]. However, no agreement on pre-emptive transplantation has been reached yet, even though patients with FA with highest risks of AML or MDS may have the indication for an upfront transplant before disease progression, as exemplified by carriers of *FANCD1/BRCA2* mutations [81]. Improved survival after transplantation has been related to age at transplant (less than 10 years), transplant performed before clonal evolution, matched family donor, and fludarabine based conditioning regimens without irradiation [81].

## 3. GATA2 Related Disorders

One of the most frequent mutations associated with germline predisposition to MDS involves the GATA binding protein 2 (*GATA2*) gene, and is transmitted with autosomal dominant inheritance. *GATA2* encodes a zinc finger transcription factor that is critical for hematopoiesis, HSC homeostasis, and lymphoid system development [29,30]. Four different phenotypes associated with a *GATA2* deficiency have been described: (i) Emberger syndrome (lymphedema and monosomy 7); (ii) MonoMAC syndrome (Monocytopenia and Mycobacterium avium complex infection); (iii) dendritic cell, monocyte, B and natural killer (NK) lymphoid deficiency (DCML); and (iv) familial MDS/AML [33,34,35]. A recent study on 508 young MDS patients identified a germline *GATA2* mutations in 7% of primary MDS cases and in 15% of advanced MDS, but not in children with MDS secondary to aplastic anemia or previous cancer therapy [31]. *GATA2* mutated patients were older at diagnosis and presented more often with monosomy 7 (identified in 70% of these patients) [31]. In patients with MDS, a *GATA2* deficiency should be suspected in case of suggestive clinical features, such as monocytopenia, infection by atypical mycobacteria, recurrent HPV infections, lymphedema or monosomy 7 [31,32]. Screening tests searching for healthy carriers should be performed in families with a case of GATA2 deficiency; however, the best management for healthy carriers is not well established. The risk of developing MDS/acute leukemia is 6% by the age of 10 years, 39% by the age of 20 years, and 81% by the age of 40 years [31]. In addition, cases of juvenile myelomonocytic leukemia and T-cell acute lymphoblastic leukemia (in addition to monosomy 7) have been described, as the oncogenic events hitting the HSCs may affect also lymphoid stem cells progenitors [31]. Allogeneic HSCT represents the only curative option for patients with *GATA2* mutation who develop MDS, and, importantly, may reverse other clinical features associated with the mutation [82].Both myeloablative and reduced intensity conditioning regimens have been successfully used as well as different donor types [30,82].

## 4. Shwachman-Diamond Syndrome

Shwachman Diamond syndrome (SDS) is a rare recessive autosomal disease, mostly caused by mutations in the *SBDS* (for Shwachman-Bodian-Diamond Syndrome) gene, also known as SBDS ribosome maturation factor, that is involved in the biogenesis of ribosomes and in mitotic spindle stabilization [36,37]. Loss-of-function mutations of *SBDS* lead to reduced levels of the SBDS protein in these patients. SDS is characterized by multiple organ involvement, including hematological disorders (usually presenting with neutropenia), bone malformations, exocrine pancreas insufficiency and cognitive impairment [38]. However, these features are not present in all patients, and hypoplastic MDS may be the first manifestation of disease [73]. In a French study on 102 patients with SDS, the cumulative incidence of MDS/AML was 18.8% at 20 and 36.1% at 30 years of age [39]. This high MDS/AML rate may be partially explained by the degree of chromosomal instability due to the malfunctioning of the SBDS protein, that is otherwise normally implied in mitotic spindle stabilization. Impaired stabilization of the mitotic spindle may then lead to the development of cytogenetic abnormalities, namely isochromosome 7 [i(7)(q)] and deletion of chromosome 20 [del(20)(q11q13)], which are partially responsible for leukemogenesis [39,83].

HSCT remains the only therapeutic approach for severe pancytopenia, MDS or leukemic transformation, and should be proposed before progression to leukemia to be more beneficial. In fact, as shown by a recent study on SDS patients developing MDS or AML, overall survival (OS) at 3 years was 51% for patients with MDS, but dropped to 11% for patients with leukemia [84].

## 5. SAMD9/SAMD9L Related Syndromes

Recent studies have identified two genes involved in the pathogenesis of MDS, located in the 7q21 chromosomal region: the sterile alpha motif (SAM) domain-9 *(SAMD9)* and *SAMD9L (SAMD9-like)* genes [40,41,42]. The proteins encoded by these genes are involved in cell proliferation control and exert an anti-proliferative function [43]. Germline gain-of-function (GOF) mutations, therefore, increase SAMD9 or SAMD9L antiproliferative effect causing pancytopenia, BMF and generally restricted growth and/or specific organ hypoplasia in non-hematopoietic tissues in an autosomal dominant fashion [44]. In hematopoietic cells, there is a selective pressure to neutralize the GOF *SAMD9/SAMD9L* mutation by the acquisition of additional somatic aberrations. Three mechanisms of reversion of *SAMD9L* mutations have been described. Two of these mechanisms are associated with the development of clonal hematopoiesis leading to the reduction of the antiproliferative effect of the mutation, namely (i) homologous recombination of the long arm of chromosome 7, replacing the mutant allele with a wild type copy; and (ii) somatic loss-of-function mutations of the pathologic allele [85]. The third mechanism of reversion of *SAMD9L* mutations associates with MDS development, and is represented by deletion of the mutant allele by total or partial loss of chromosome 7 [monosomy 7/del (7q)/der(1;7)] causing evolution to MDS [44,86]. For these reasons, patients with pediatric MDS with monosomy 7, del 7q and der(1;7), as well as adult MDS patients with these chromosomal aberrations and a suspected genetic predisposition, should be tested for *SAMD9* and *SAMD9L* mutations on tissues different from blood [40].

The phenotype associated with *SAMD9* mutations (MIRAGE syndrome) appears to be more severe, due to an elevated risk of early-onset MDS with monosomy 7 and an elevated childhood mortality due to infections, anemia and/or hemorrhages [41]. By contrast, *SAMD9L* mutations, initially identified in ataxia-pancytopenia syndrome (ATXPC) and in myelodysplasia and leukemia syndrome with monosomy 7 syndrome (MSML7), seem to be associated with milder non-hematological disease manifestations like cerebellar dysfunction, beside cytopenia, immunodeficiency and predisposition to MDS, with a variable penetrance explained by hematopoietic somatic revertant mosaicism or compensatory effect of germline mutations [43,44].

Concerning treatment before transformation to MDS, if allogeneic HSCT is indicated because of cytopenia, non-myeloablative regimens similar to the ones used for Fanconi’s anemia or GATA2 deficiency syndrome should be preferred. In fact, in both ATXPC and MIRAGE syndromes, there is a reduced risk of rejection due to a lower competitiveness of recipient HSC and a decreased number and function of monocytes and NK, and a high risk of transplant related neurological toxicity associated to myeloablative regimen [40,87]. However, once transformation to MDS has occurred, a careful risk-benefit analysis should be carried out to select the best conditioning regimen pre-HSCT [88].

## 6. Diamond-Blackfan Anemia

Diamond–Blackfan anemia (DBA) is a rare congenital hypoplastic anemia characterized by a block in the maturation of the erythroid progenitors, with half of the cases presenting with a variety of congenital malformations [50]. DBA is most frequently due to a sporadic mutation (55%) in genes encoding several different ribosomal proteins, although there are many cases showing a family history of the disease with varying phenotypes. DBA is thus a polygenic disease with mutations in 20 of the 80 ribosome protein (RP) genes that code for the ribosome complex. Interestingly, deletions in six of the 20 identified genes, namely *RPS19, RPL5, RPS26, RPL11, RPL35a*, and *RPS24*, account for 70% of all DBA cases [49]. The major unresolved questions in DBA remain how a defect in RP is responsible for a specific defect in erythropoiesis, and why there is a variable penetrance of the same mutation among different carriers. Patients with DBA typically present with severe macrocytic hyporigenerative anemia in infancy. Short stature is common in DBA; other physical features are bone, genitourinary and heart malformations. However, the spectrum of clinical manifestations of DBA is often wide, and some patients may not develop anemia until later in life. Heterozygous mutation carriers may lack a clinical phenotype due to incomplete genetic penetrance and phenotypic expressivity [14].

The current standard of care for DBA includes corticosteroids, which improve anemia in 80% of cases; however, prolonged corticosteroid treatment is problematic for many patients and only 40% remain on treatment for a considerable period of time. With existing treatments, the OS of patients, as reported by the DBA Registry of North America, is 75% at 40 years of age [51]. A major component of morbidity and mortality is due to long term malignancies, as DBA is recognized as a cancer predisposition syndrome with an observed to expected ratio for all cancers of 5.4. Among the most common late malignancies, there are MDS and AML, and the only curative treatment for these hematologic conditions is HSCT [22,51].

## 7. *RUNX1*, *ANKRD26* and *ETV6* Related Familial Thrombocytopenia

A subset of patients with familial thrombocytopenia are at increased risk of developing myeloid neoplasms during their life time, particularly those with germline autosomal dominant mutations in the *RUNX1*, *ANKRD26*, and *ETV6* genes [45]. These three germline predisposition disorders have a prevalence rate of 3%, 18%, and 5%, respectively, among inherited thrombocytopenia [46]. Patients may present with mild to moderate isolated thrombocytopenia, normal platelet size, megakaryocytic atypia and BM features that may overlap with idiopathic thrombocytopenic purpura (ITP) or sporadic MDS, thus leading to a risk of misdiagnosis [46].

MDS and AML are reported in about 40% of patients with *RUNX1* germline mutations and in 8% of patients with *ANKRD26* related thrombocytopenia, whereas 23% of the 73 patients with *ETV6* familial thrombocytopenia described so far had hematological malignancies [47,48]. Of note, in 2016 the World Health Organization classification included myeloid malignancies arising from germline mutations in *ANKRD26*, *RUNX1*, and *ETV6* into a new category defined as “Myeloid neoplasms with germline predisposition and pre-existing platelet disorders” [1]. Once a diagnosis of inherited thrombocytopenia with germline mutation in *RUNX1, ANKRD26,* or *ETV6* is established, close and long-term surveillance is mandatory given the lifelong increased risk of developing hematologic malignancies.

## 8. Severe Congenital Neutropenia

Severe congenital neutropenia (SCN) comprises a heterogeneous group of hematological diseases that are characterized by a defect in granulocytopoiesis and increased risk for recurrent and often life-threatening infections [52]. SCN is most commonly caused by autosomal dominant mutations in the *ELANE* gene, which encodes neutrophil elastase, and autosomal recessive mutations in *HAX1*, which contributes to the activation of the granulocyte-colony stimulating factor (G-CSF) signaling pathway [54]. The cumulative incidence of MDS/leukemia after 15 years on treatment with G-CSF is 20 to 30% [53]. Molecular events that lead to clonal evolution and malignant transformation include acquired mutations in the *CSF3R* gene (encoding the G-CSF receptor) and subsequently in other leukemia-associated genes (such as *RUNX1*) [53]. Daily subcutaneous G-CSF administration leads to a substantial increase in blood neutrophils counts and to a reduction of infections, but HSCT is the only curative treatment for SCN [89]. Close clinical observation, including yearly BM evaluations to detect chromosomal abnormalities such as trisomy 21 and monosomy 7 as well as somatic leukemogenic mutations, is highly recommended [90].

## 9. Cytogenetic Features of IBMFS

There is a number of recurrent cytogenetic alterations frequently seen in MDS predisposition syndromes. Monosomy 7 and del(7q) are the most common, in particular in DC, FA, GATA2 and SAMD9/SAMD9L disorders, SDS, and SCN; other cytogenetic alterations include +1q, 3q amplification, +13q in FA, and del20q and iso(7)q in SDS. However, up to date, the characteristics and roles of acquired cytogenetic alterations, together with their interaction with somatic mutations in IBMFS, remain poorly understood. In fact, it has been suggested that acquired somatic mutations may precede cytogenetic clonal evolution and might help in defining the patient prognosis [91].

### Monosomy 7 in IBMFS

The complete loss of chromosome 7 or a deletion of its long arm is one of the most common cytogenetic abnormalities in pediatric and adult myeloid malignancies, and may occur alone or within a complex karyotype. Analysis of MDS/AML patient outcomes suggests that −7/del(7q) carries a poorer prognosis compared to other cytogenetic abnormalities, even if the partial loss of chromosome 7 has a more favorable effect on outcome than a total loss [92]. However, according to recent evidence, the impact on survival of the two chromosome 7 abnormalities may be modulated by the concomitant presence of additional gene mutations [93]. Interestingly, −7/del(7q) may be found in 17% of MDS and AML arising in a context of IBMFS, in particular in 13% of FA, 12% of DC (the only chromosomal alteration reported), and 20% of GATA 2 deficiency [92], and it is associated with poor survival [92].

## 10. HSCT in MDS Arising in IBMFS

HSCT is currently the only curative treatment for MDS, with or without genetic predisposition. HSCT is the treatment of choice for most patients with BMF and is preferentially carried out with radiation-free reduced intensity conditioning (RIC) regimens. BM is the preferred graft in T-repleted transplants, because peripheral blood is associated with higher risk of graft-versus-host disease (GVHD) and second malignancy [12,94,95].

Patients with IBMFS should undergo extensive clinical and laboratory evaluation before and after HSCT. Clinical manifestations are heterogenous and have variable penetrance within affected members of the same family, and screening of family members is essential in order to consider them, or exclude them, as potential HSCT donors. As IBMFS can impact organs other than the BM, the pre-transplant evaluation of these patients, and of potential family donors, requires a multidisciplinary team [90]. The geneticist must be involved in counseling the patient and the family members. A visual, hearing, endocrine, nutritional, and neuropsychologic evaluation may be needed in most patients. Moreover, patients should undergo oral examination performed by a dentist, detailed skin examination by a dermatologist in the case of FA and TBD, and other evaluations as needed (such as gastrointestinal endoscopy or nasolaryngoscopy screening) [90]. The hematologic evaluation should include disease reassessment (single or multilineage cytopenias, MDS, AML), complete blood count, as well as dosage of erythrocyte adenosine deaminase, fetal hemoglobin and α-fetoprotein levels. Fluorescent in situ hybridization (FISH) and cytogenetics should be performed, particularly in FA. In case of prior use of androgens, signs of virilization, growth problems, and liver dysfunction should be searched. In case of prior use of steroids, patients should be evaluated for the presence of Cushing’s syndrome symptoms: hyperglycemia, hypertension, metabolic syndrome, avascular necrosis, and adrenal insufficiency. Finally, a close monitoring for any type of cancer before and after HSCT is very important in this population [90].

## 11. Pitfalls in Detecting IBMFS in Adult Patients: A Case Report

A 27-year-old Moroccan man was referred to our clinic for persistent neutropenia. His past medical history included a left brachial plexus injury at the time of delivery, and a condition of epilepsy diagnosed two years before. He had six siblings in good health, whereas both parents had died, the father of unknown cause and the mother of leukemia not otherwise specified. The patient had no striking physical abnormalities, height and weight were on average, and physical examination did not reveal any palpable lymphadenopathy or hepatosplenomegaly. He presented with moderate leukopenia with absolute neutropenia (WBC 3.200/μL with neutrophils 960/μL), and with normal levels of hemoglobin, platelets, vitamin B12 and folic acid. Table 2 summarises the patient’s characteristics.

### 11.1. What Should We Rule Out?

**Figure 2:**
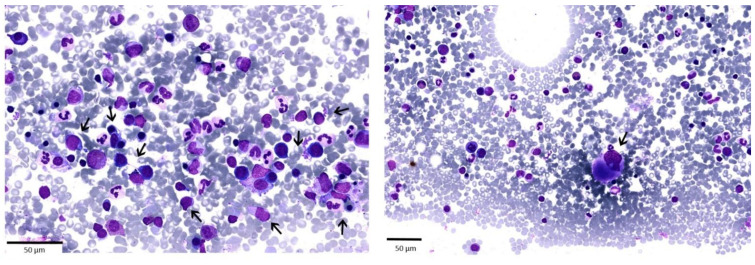
Bone marrow aspirate of the patient presenting with neutropenia and carrying *TERT* mutations. The bone marrow smear (May-Grünwald-Giemsa) staining shows morphological abnormalities in erythroid lineage (cytoplasmic vacuolization, cytoplasmic bridges, pyknosis, incomplete hemoglobinization); myeloid dysplastic features (increased myeloblasts, neutrophil hypogranulation) and megakaryocytes dysplasia (hypolobulated megakaryocytes). Black arrows point to the main dysplastic features.

The young age and the North-African descent may suggest ethnic neutropenia; however, the patient reported normal blood counts in the past. A complete autoimmunity pannel was carried out, including anti-neutrophil antibodies, along with viral screening for HBV, HCV, HIV, and CMV which turned out negative. The peripheral blood smear showed absolute neutropenia with hypogranulated forms, whereas red cells and platelets were normal. A BM aspirate was performed, showing a normocellular marrow with trilineage dysplasia, more pronounced in the myeloid series, with no excess of myeloid blasts (2%) and some hypolobated megakaryocytes (Figure 2). Flow cytometry and the BM biopsy confirmed the dysplastic features and the low blast count, whereas cytogenetics showed a normal male karyotype. The diagnosis was consistent with MDS with multilineage dysplasia (MDS-MLD) at IPSS Intermediate-1 Risk and R-IPSS very low risk [2,96]. Despite the low risk of progression, the young age of the patient prompted us to perform NGS analysis using a panel of 50 genes commonly mutated in myeloid diseases (see Supplementary Materials Table S1). No somatic mutation was detected by this approach.

### 11.2. Should the Diagnostic Process Stop Here?

The normal karyotype and the absence of somatic mutations are a rare event in MDS, since almost 50% of patients carry a cytogenetic driver and 90% of patients carry at least one somatic mutation of genes involved in myeloid malignancies and included in the NGS panel that had been applied to the patient [5]. Several reasons prompted further testing in order to assess the presence of an inherited genetic predisposition to MDS: (i) a driver of clonal hemopoiesis had not been found; (ii) the patient’s age fell outside the expected age range for MDS; and (iii) the family history of the patient included a first degree relative with a hematological malignancy.

### 11.3. Which Tests Should Be Run to Exclude IBMFS?

We performed chromosomal breakage studies in response to diepoxybutane and mitomycin C and cellular cycle testing to exclude mutations in the DNA repair machinery, which turned out normal. Then we measured telomere length, which turned out below the 33rd percentile, suggesting an inherited telomeropathy. On these grounds, we subsequently screened the patient for DC and found two *TERT* variants in double heterozygosity. The first *TERT* variant was c.835G > A, a variant highly prevalent in the Genome Aggregation Database (frequency 0.01), although enriched almost 2 fold in the aplastic anemia and MDS cohorts, and thought to confer a mild predisposition to MDS. The second *TERT* variant was c.833C > T, a variant of uncertain significance already described once in ClinVar and predicted to be pathogenic or benign by different informatic tools. Both variants were confirmed to be germline. We subsequently assessed the presence of extra-hematological organ damage of liver and lung, which showed no significant impairment.

**Figure 3:**
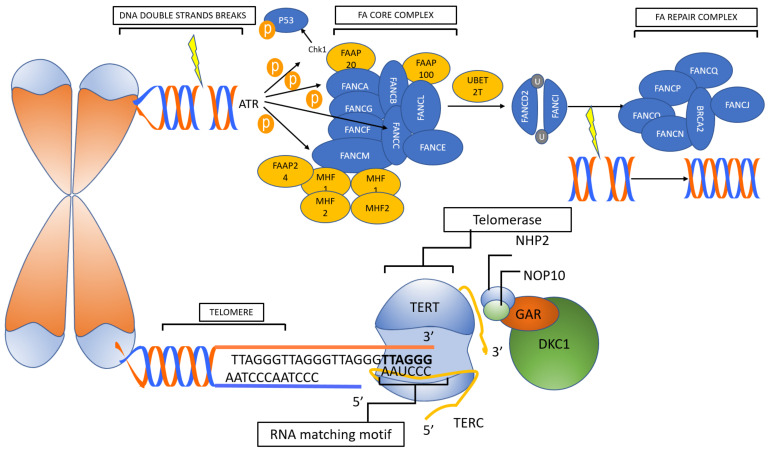
Telomerase function and DNA damage response system. Telomerase is the ribonucleoprotein responsible for the preservation of telomeres. Under normal condition, telomerases are able to counterbalance the physiological nucleo-tide loss of the chromosomal telomeric portion, and therefore prevents chromosome end-to-end fusions and rearrange-ments. In parallel, DNA damage response manages lesions and mutations occurring in non-telomeric portions of the DNA double strands. Primary telomeropathies are directly related to telomerase dysfunction, whereas secondary telom-eropathies are related to the misfunction of the DNA damage response proteins. For details, see text section on telomer-opathies. FA, Fanconi anemia.

The penetrance of the clinical phenotype associated with *TERT* mutations seems to be lower than that associated with *TERC* mutations, with a higher frequency of asymptomatic or mildly affected mutation carriers and a more heterogeneous disease. This could be due to the fact that fewer *TERT* mutations are null, leading to a higher residual telomerase activity in *TERT* mutated cells as compared to *TERC* mutated cells [97]. Figure 3 describes telomerase function and DNA damage response system.

### 11.4. How Is the Detection of a Telomeropathy Going to Change the Approach to the Patient?

At present, the patient does not require treatment. However he has an expected OS of 9 years according to r-IPSS, and due to the natural history of disease he will likely be a candidate for HSCT. Out of the six siblings of the patient, only one sister was available for genetic testing. She was in good health and her blood count was normal. She was screened for the presence of germline *TERT* mutations, which were absent, and afterwards she was HLA-typed and resulted identical. The patient is currently asymptomatic with no signs of progression and with stable blood count.

Several studies have shown that, in families with *TERT* mutations, age specific telomere lengths are shorter in later generations, a phenomenon known as genetic anticipation. This implies that a child with BMF or DC inherits the mutated gene and pre-shortened telomeres from the affected parents. Conceivably, both a mutated *TERT* gene and pre-shortened telomeres are required for subsequent disease development. It is therefore crucial to assess the patient and his family members for *TERT* mutations and provide a correct genetic counseling, even for future generation [97].

## 12. Discussion and Conclusions

Detecting a germline MDS predisposition disorder has profound implications on patient management and treatment. Prompt diagnosis of these disorders allows surveillance to detect early signs of disease progression and, consequently, a timely scheduling of HSCT before progression to leukemia, which often carries a poor prognosis. The definitive screening and diagnostic approach for some conditions of MDS predisposition is genetic testing, as exemplified by the case of *SAMD9/SAMD9L* and *GATA2* mutations, whereas, for other conditions, functional tests are available and are preferred for screening, as exemplified by the case of FA and DC. Figure 1 summarizes the different screening tests for specific conditions.

Although IBMFS are typically diagnosed in childhood or adolescence, an increasing number of patients may present to adult hematologists with atypical presentations. Careful consideration should be given to the patient’s history and physical findings, and family history needs to be investigated to detect other relatives with IBMFS, hematologic malignancies, solid tumors, cytopenia or bleeding phenotypes, frequent or unusual infections, early deaths or miscarriages, any toxicities with therapy, and pulmonary or hepatic complications [90,98] (Figure 1). Many disorders are associated with intrauterine growth retardation, so birth weight and prenatal history should be investigated. It is useful to revisit and update the family history over time as complications or malignancies may develop during patient follow-up.

Almost all types of IBMFS result from single germline mutations, whereas adult MDS harbor multiple mutations with complex clonal architecture [4,99]. Although there are a number of caveats in the accurate interpretation of NGS results when a patient’s presentation is phenotypically ambiguous, NGS can often outperform candidate gene testing and ancillary testing both in efficiency and cost [11,100,101]. Whenever possible, genetic testing should be performed on constitutional tissue, preferably on skin fibroblasts, in order to exclude somatic mutations and to avoid false-negatives due to peripheral blood somatic mosaicism.

Importantly, the phenotypic spectrum of MDS predisposition syndromes remains largely undefined, and, for many of the syndromes, incomplete penetrance and variable expressivity may limit the ability to diagnose these disorders. Indeed the diagnosis of genetic predisposition to MDS in individuals without clear syndromic features, as shown by the clinical case here illustrated, may be difficult and the integration of the different tests together with the family history is crucial to orient the diagnosis. It is of paramount importance to relay on a network of expertise which should include a geneticist, and in some cases a pediatrician, to be able to perform all the required tests and to be sure to rule out all the known IBMFS.

## Figures and Tables

**Table 1:** Most common germline disorders associated with MDS.

Syndromes	Gene	Inheritance	Cellular Function	Associated Phenotype	Evolution to MDS/AML
DC/Telomeropathy	*DKC1* [18]	X-linked	Telomere maintenance	Mucocutaneous features (nail dystrophy, skin pigmentation abnormalities, oral leukoplakia) Idiopathic pulmonary fibrosis Liver diseases Immunodeficiency/immune dysregulation Endocrinopathies Osteoporosis, dental abno malities, short stature CNS abnormalities/cerebellar hypoplasia Secondary cancer (oral and gastrointestinal squamous cell carcinoma) [19,20,21,22]	Cumulative incidence of evolution to MDS i creases with age, with a prevalence of 13% in non-transplanted patients [22]
	*TERT, TERC,* *TINF2, RTEL1* *ACD/TPP1,* *PARN, NAF1* *STN1*	AD			
	*TERT, WRAP53* *NOLA3, NOLA2* *TCB1, RTEL1* *CTC1, CD/TPP1* *PARN, NHP2* *NOP10* [23]	AR			
FA	*FANC* (*A, C, D1,* *D2, E, F, G, I, J, L,* *M, N, O, P, Q, R,* *S, T, U, W*)	AR	DNA Repair pathway	Short stature, café au lait, spots and hyper/hypopigmentation, abnormal thumbs, absent radii, microcephaly, micro- ophthalmia, structural renal/urogenital, cardiac malformations abnormalities/malformations, endocrinopathies, hypogonadism, squamous cell carcinoma: oral, gastrointestinal, genitourinary. FANCD1/BRCA2 Subtype: solid tumors and ALL [24,25]	Cumulative incidence of evolution to MDS/AML of 33% by 40 years old [25,26,27]
	*FANCB* [28]	X-linked			
Emberger syndrome MonoMAC syndrome DCML	*GATA2* [29,30]	AD	Transcription factor	Emberger syndrome: lymphedema, sensorineural hearing loss and monosomy 7 MonoMAC syndrome: monocytopenia and Mycobacterium avium complex infection DCML: susceptibility to mycobacterial, fungal, viral infections; warts; molluscum; pulmonary alveolar proteinosis [31,32]	Cumulative risk of evolution to MDS/AML: 6% at the age of 10 years, 39% at the age of 20 years, and 81% at the age of 40 years [33,34,35]
SDS	*SBDS* [36,37]	AR	Biogenesis of ribosomes and mitotic spindle stabilization	Short stature, exocrine pancreatic dysfunction, pancreatic lipomatosis/atresia, skeletal dysplasia, osteopenia, eczema, transient transaminitis/hepatomegaly in early childhood, dental anomalies, immunodeficiencies, endocrinopathies, neurocognitive and other variable congenital anomalies [38]	Cumulative risk of evolution to MDS/AML: 18.8% at 20 years and 36.1% at 30 years of age [39]
MIRAGE syndrome ATXPC/MLSM7	*SAMD9* *SAMD9L* [40,41,42]	AD	Proliferation control	MIRAGE syndrome: cytopenias, immunologic abnormalities, short stature, a renal hypoplasia, invasive bacterial infections, gastrointestinal (chronic diarrhea, genitourinary abnormalities, delay of developmental milestones, intrauterine growth restriction ATXPC: ataxia, cerebellar hypoplasia, invasive bacterial infections, alveolar proteinosis, cytopenia [43,44]	No data on cumulative risk of evolution to MDS/AML
Familiar MDS associated with thrombocytope-nia	*RUNX1,* *ANKRD26,* *ETV6* [45]	AD	Transcription factor	Thrombocytopenia, platelet dysfunction [46]	Prevalence of MDS and AML is of about 40% in patients with *RUNX1*, of 8% in patients with *ANKRD26* and of 23% in patients with ETV6 [47,48]
DBA	*GATA1, RPL5,* *9, 11, 15, 18, 26,* *27, 31, 35, 35a,* *RPS7, 10, 15a, 17,* *19, 24, 26, 27, 28,* *29* [49]	AD	Ribosomopathy	Short stature, severe macrocytic hyporigenerative anemia in infancy, facial dysmorphisms, radial ray anomalies, skeletal anomalies, genitourinary and heart malformations. Neutropenia and immunodeficiencies associated with RPL35a [50]	Cumulative risk of evolution to AML of 2% by 45 years [51]
Severe congenital neutropenia	*ELANE* [52]	AD	ELANE encodes for neutrophil elastase	Osteopenia	Cumulative risk of evolution to MDS/AML of 22% after 15 years for *ELANE* [53]
	*GFI1*			Lymphopenia	
	*HAX1*	AR		Seizures, neurologic abno malities [54]	
	*G6PC3*			Structural heart disease, urogenital anomalies, prominent veins, deafness, skeletal anomalies, immune dysregulation, colitis, poor growth, thrombocytopenia [53]	
	*JAGN1* [54]			Skeletal, dental anomalies	

Abbreviations: AD, autosomal dominant; AR, autosomal recessive; ATXPC, ataxia-pancytopenia syndrome; DBA, Diamond-Blackfan anemia; DC, Dyskeratosis congenita; DCML, dendritic cell, monocyte, B and natural killer (NK) lymphoid deficiency; FA, Fanconi anemia; MDS, myelodysplastic syndromes; MLSM7, myelodysplasia and leukemia syndrome with monosomy 7 syndrome; SDS, Schwachman Diamond syndrome.

**Table 2:** Main characteristics of the patient and diagnostic findings.

Main Characteristics of the Patient and Diagnostic Findings	
Age (years)	27
Sex	Male
Congenital abnormalities	None
Comorbidities	Left brachial plexus injury; Epilepsy
Family history	Father†; Mother† (leukemia)
Complete blood count	WBC 3.200/µL Neutrophils 850 × 10^6/µL Lymphocytes 1700 × 10^6/µL Monocytes 500 × 10^6/µL Eosinophils 150 × 10^6/µL Basophils 100 × 10^6/µL Hemoglobin 14.1 g/dL Platelets 196.000/µL Blasts < 2%
ANA	Negative
HBV, HCV, HIV	Negative
Bone marrow aspirate	Trilinear dysplasia, blasts 3%
Karyotype	46, XY [20]
NGS analysis on 52 genes commonly mutated in MDS	No mutation
DEB and Cell cycle test	Negative
Telomere length	<33° percentile
Somatic and germline testing for DC associated mutations	c.835G > A p.Ala279Thr and c.833C > T p.Pro278Leu mutation in heterozygosity in *TERT* gene
Hepatic elastometry	Absence of steatosis or fibrosis
Pulmonary CT scan	No signs of fibrosis

Abbreviations: ANA, Anti-neutrophils antibodies; CT, computed tomography; HBV, Hepatitis B Virus; HCV, Hepatitis C Virus; HIV, human immunodeficiency virus; NGS, Next Generation Sequencing; WBC, White Blood Cell.

## Supplementary Materials

Supplementary Materials can be found at https://www.mdpi.com/1422-0067/22/5/2525/s1, Table S1: NGS analysis using a panel of 50 genes commonly mutated in myeloid diseases.
